# Novel Strategies for the Treatment of Chondrosarcomas: Targeting Integrins

**DOI:** 10.1155/2013/396839

**Published:** 2013-12-30

**Authors:** Jui-Chieh Chen, Yi-Chin Fong, Chih-Hsin Tang

**Affiliations:** ^1^Graduate Institute of Basic Medical Science, China Medical University, No. 91, Hsueh-Shih Road, Taichung 40402, Taiwan; ^2^National Institute of Cancer Research, National Health Research Institutes, Miaoli 35053, Taiwan; ^3^School of Chinese Medicine, College of Chinese Medicine, China Medical University, Taichung 40402, Taiwan; ^4^Department of Orthopedic Surgery, China Medical University Hospital, Taichung 40402, Taiwan; ^5^Department of Pharmacology, School of Medicine, China Medical University, Taichung 40402, Taiwan; ^6^Department of Biotechnology, College of Health Science, Asia University, Taichung 41354, Taiwan

## Abstract

Chondrosarcomas are a heterogeneous group of malignant bone tumors that are characterized by the production of cartilaginous extracellular matrix. They are the second most frequently occurring type of bone malignancy. Surgical resection remains the primary mode of treatment for chondrosarcomas, since conventional chemotherapy and radiotherapy are largely ineffective. Treatment of patients with high-grade chondrosarcomas is particularly challenging, owing to the lack of effective adjuvant therapies. Integrins are cell surface adhesion molecules that regulate a variety of cellular functions. They have been implicated in the initiation, progression, and metastasis of solid tumors. Deregulation of integrin expression and/or signaling has been identified in many chondrosarcomas. Therefore, the development of new drugs that can selectively target regulators of integrin gene expression and ligand-integrin signaling might hold great promise for the treatment of these cancers. In this review, we provide an overview of the current understanding of how growth factors, chemokines/cytokines, and other inflammation-related molecules can control the expression of specific integrins to promote cell migration. We also review the roles of specific subtypes of integrins and their signaling mechanisms, and discuss how these might be involved in tumor growth and metastasis. Finally, novel therapeutic strategies for targeting these molecules will be discussed.

## 1. Chondrosarcomas

Chondrosarcomas are a heterogeneous group of malignant bone tumors with diverse histopathology and clinical behavior, which are characterized by the production of cartilage matrix. They are the second most common type of skeletal malignancy after osteosarcomas [1]. Chondrosarcomas are usually found within flat bones; the pelvis and femur are two common sites of involvement, although any bone may be affected [2, 3]. These malignant cartilaginous tumors may either arise *de novo* or develop from pre-existing benign lesion (e.g., enchondromas and osteochondromas), termed primary (or conventional), and secondary chondrosarcomas, respectively. Tumors can arise in both skeletal (central) and extraskeletal (peripheral) locations [4]. The majority of cases are primary central chondrosarcomas; together, primary central and secondary peripheral chondrosarcomas constitute approximately 85% of all chondrosarcomas. Other specialized types of chondrosarcoma, such as dedifferentiated, clear cell, and mesenchymal chondrosarcomas, account for the remaining 10%–15% of cases [3, 5].

Chondrosarcomas are classified into three histological grades (grades 1–3), based on the extent of cellularity, nuclear atypia, nuclear staining (hyperchromasia), muco-myxoid matrix changes, and increased vascularization [6–9]. Approximately 90% of conventional chondrosarcomas are grade 1 or 2, which have an indolent clinical course, low metastatic potential, and good prognosis; the remaining 5–10% are grade 3 tumors, which have high metastatic potential and are associated with poor outcomes [3, 10, 11]. For chondrosarcomas, prognosis is strongly correlated with histological grade, as well as with the adequacy of the resection margins [12, 13]. Low-grade chondrosarcomas show little cellularity and an abundant matrix that resembles hyaline cartilage. These tumors rarely metastasize and are therefore often managed with intralesional curettage and resection. In contrast, high-grade conventional chondrosarcomas are highly cellular, with little or no cartilaginous matrix. High-grade tumors often metastasize, leading to lethality in most cases; for these, radical excision margins, or even amputation, may be recommended [3].

Chemotherapy and radiotherapy are largely ineffective for treating chondrosarcomas, due to the slow growth, abundant extracellular matrix (ECM), low percentage of dividing cells, and poor vascularity of these tumors [14–17]. Moreover, some studies indicate that chondrosarcoma cells can express multidrug-resistance gene products, such as P-glycoprotein, thereby reducing the absorption of drugs, and giving rise to chemotherapy resistance [18, 19]. Surgical resection remains the primary mode of treatment for chondrosarcomas. In a minority of patients, local recurrence or metastasis occurs, and can result in death; this is more prevalent in those with high-grade tumors [3, 16]. The above features make the clinical management of chondrosarcomas particularly challenging, and new therapeutic strategies are urgently needed. One type of approach focuses on inhibiting the processes of metastasis and invasion, and may facilitate the development of effective adjuvant therapy. Integrins have been considered potential therapeutic targets because they are exposed on the cell surface and are sensitive to pharmacological blockade.

## 2. Characteristics of Integrins

Integrins are a family of heterodimeric transmembrane glycoproteins that are found on nearly all cells, where they function as adhesion receptors, mediating dynamic cell-cell and cell-extracellular matrix interactions. Through these interactions, integrins play critical roles in cancer cell migration, invasion, and metastasis—processes that contribute to tumor progression [20]. To date, at least 24 unique integrin heterodimers have been identified. These heterodimers are formed from various combinations of 18 *α*-subunits and 8 *β*-subunits, which interact noncovalently. Each integrin subunit consists of a large extracellular domain, a single type I transmembrane domain, and a short intracellular cytoplasmic tail domain [21]. The ligand-binding site of an integrin heterodimer lies at the interface between the two subunits. Their cytoplasmic domains form connections with the cytoskeleton, enabling integrins to serve as a link between the ECM and the cytoskeleton.

Ligand specificity is determined by the extracellular domain of the integrins. Five main groups have been identified: arginine-glycine-aspartate (RGD)-binding, the *α*4 family, laminin-binding, I-domain collagen-binding, and leukocyte adhesion integrins. Approximately one third of integrins have binding sites for the RGD sequence, which is found on many ECM proteins. Although the RGD sequences within collagen and laminin are not normally exposed, denaturation or cleavage of these proteins may result in exposure of the RGD sequence and subsequent integrin binding. Generally, *α*4 integrins recognize the leucine-aspartic acid-valine (LDV) tripeptide, collagen-binding integrins recognize a triple helical collagen peptide containing the glycine-phenylalanine-hydroxyproline-glycine-glutamate-arginine (GFOGER) motif [22, 23]. Integrins do not simply act as adhesion molecules; they can also function as bidirectional signaling molecules, controlling a variety of cell functions such as proliferation, differentiation, survival/apoptosis, cell shape, polarity, or motility, as well as gene expression [21].

## 3. Integrin-Dependent Signaling

Although integrins lack intrinsic kinase activity, signal transduction can be induced by the assembly of signaling complexes on the cytoplasmic domains of integrin subunits. Through interactions of their cytoplasmic domains with a wide variety of adaptor proteins, integrins are able to deliver signals into the cell in response to extracellular cues (“outside-in” signaling). In addition, some cytoplasmic interactions can induce conformational changes in integrin molecules. This can affect their activation state by modulating their binding affinity for extracellular ligands (“inside-out” signaling) [24–26]. In the inactive or low-affinity state, integrins are in a “bent” conformation, with the transmembrane and cytoplasmic domains close together; this impedes ligand engagement and maintains the low-affinity state. The binding of talins and kindlins to their respective binding regions of the *β* integrin cytoplasmic tails induces conversion from the bent to the extended conformation. This separates the cytoplasmic and transmembrane subunits and results in a shift to the activated or high-affinity state. When activated integrins bind to ligands, they cluster at the plasma membrane. This clustering promotes intracellular signaling, resulting in the formation of tight focal adhesions, actin cytoskeletal assembly, and activation of multiple downstream signaling pathways that influence a variety of cellular functions [27–29]. Precise regulation of ligand binding affinity is therefore critical for proper integrin function.

The canonical view has been that ECM ligands bind to their cognate integrins and initiate signaling via specific pathways, to give rise to distinct cell responses. However, accumulating evidence reveals that several integrins are able to crosstalk with oncogenic signal transducers, such as ErbB, Ras, and Src, to promote tumorigenesis [30–34]. Cooperative signaling between integrins, growth factor receptors, and cytokine receptors has also been implicated in tumor progression [35–41]. Upon ligand binding, integrins may trigger cells to secrete growth factors and/or cytokines, which in turn can bind to their receptors in an autocrine or paracrine manner to induce further signaling. For example, the activation of integrin *α*v*β*3 can trigger phosphorylation of p66 Shc; this has been shown to upregulate the expression of vascular endothelial growth factor (VEGF), leading to tumor growth and angiogenesis in human prostate and breast cancer cells [38]. In pancreatic cancer cells, the *α*6*β*1 and *α*3*β*1 integrins interact with laminin-1 to mediate cell migration; this process involves the upregulation of CXC chemokine receptor 4 (CXCR4) and IL-8 expression in response to the chemokine ligand CXCL12, also known as stromal cell-derived factor-1 (SDF-1) [42]. Integrin activation of growth factor receptors, through collaborative mechanisms, has also been reported to induce downstream signaling [43]. Alternatively, both growth factor and chemokine signaling may regulate integrin function by directly controlling integrin expression levels.

## 4. Regulation of Integrin Gene Expression 

A number of growth factors and chemokines/cytokines have been found to regulate the expression of integrins in many malignancies, indicating a critical role in cancer progression. For example, heparin-binding EGF-like growth factor has been observed to increase integrin expression in human breast and esophageal cancer cells [67, 68]. Binding of the CXCL12 chemokine to its receptor (CXCR4) may regulate tumor dissemination in prostate tumor cells by enhancing expression of *α*v*β*3 integrins [40]. More recently, a study demonstrated that CXCL12 strongly induced *α*v*β*6 integrin expression in ovarian cancer, leading to enhanced urokinase plasminogen activator (uPA)-mediated ECM degradation and cell invasion [69]. In human osteosarcoma cells, the CCL5/CCR5 axis can induce increased expression of *α*v*β*3 integrin via the MEK, ERK, and NF-*κ*B pathways, thereby contributing to cell migration [70]. The pro-inflammatory cytokine interleukin-1*α* (IL-1*α*) can induce selective upregulation of *α*6*β*1 integrin in pancreatic cancer cells and has been suggested to modulate tumor aggressiveness [71, 72]. Transforming growth factor-*β*1 (TGF-*β*1), a multifunctional cytokine, can promote human hepatocellular carcinoma (HCC) cell invasion by stimulating *α*3 integrin expression [44]. Another study found that TGF-*β*1 treatment promotes gastric carcinoma cell adherence by increasing *α*3 integrin levels [67].

In human chondrosarcoma cells, numerous studies have shown that growth factors, chemokines/cytokines, and other inflammation-related molecules can control the expression of specific integrins to promote cell migration. Among the growth factors, insulin-like growth factor-I (IGF-I) is able to enhance the migration of chondrosarcoma cells by increasing *α*v*β*1 integrin expression, through the IGF-I receptor/PI3K/Akt/NF-*κ*B pathway [45]. Brain-derived neurotrophic factor (BDNF) is a small protein from the neurotrophin family of growth factors whose expression has been associated with disease status and outcomes in various cancers. Recent research has shown that BDNF enhances the migration of chondrosarcoma by increasing *β*5 integrin expression, through the TrkB receptor, PI3K, Akt, and NF-*κ*B pathways [46] (Table 1).

Interleukin-8 (IL-8), a chemokine also known as CXCL8, interacts with the CXCR1 and CXCR2 receptors to activate PI3K and Akt pathways, and induce AP-1 activation. In human chondrosarcoma cells, IL-8 induced upregulation of *α*v*β*3 integrin expression and increased cell migration [47]. Metastasis, particularly to the lungs, is often observed with high-grade chondrosarcomas. Interestingly, the CXCL12/SDF-1 chemokine, which is constitutively secreted by human lung epithelium cells, has been shown to enhance the invasiveness of chondrosarcoma cells by increasing *α*v*β*3 integrin expression, through the CXCR4/ERK/NF-*κ*B pathway. It has also been observed that the expression of CXCR4 in human chondrosarcoma tissues and chondrosarcoma cell lines is higher than in normal cartilage and in human chondrocytes. This could potentially account for the homing of chondrosarcoma cells to the lung [48] (Table 1).

Accumulating evidence suggests that fat tissue can function as an endocrine organ, producing and secreting a variety of bioactive substances that are referred to as adipocytokines or adipokines. Most adipocytokines are pro-inflammatory cytokines, such as tumor necrosis factor-*α* (TNF-*α*). Various adipocytokines, including TNF-*α*, leptin, and adiponectin, have been reported to enhance chondrosarcoma cell migration by increasing the expression of specific integrins. A range of signaling pathways are involved. For example, TNF-*α* and leptin were found to increase *α*v*β*3 integrin expression, through their effects on the MEK/ERK/IKK*α*/*β*/NF-*κ*B and the OBR1/IRS-1/PI3K/Akt/NF-*κ*B pathways, respectively [49, 50]. Adiponectin is a member of the C1q and tumor necrosis factor superfamily, and structurally resembles TNF-*α*. Adiponectin can promote migration of human chondrosarcoma cells by upregulating *α*2*β*1 integrin, via AdipoR-, AMPK-, p38-, IKK*α*/*β*-, and NF-*κ*B-dependent pathways [51]. Macrophage migration-inhibitory factor (MIF), a pro-inflammatory cytokine involved in macrophage migration and activation, is able to enhance the migration of chondrosarcoma cells by increasing *α*v*β*3 integrin expression, mediated via PI3K/Akt/NF-*κ*B signaling [52] (Table 1).

The transforming growth factor-*β* (TGF-*β*) superfamily includes the prototypical member TGF-*β*, and numerous others, such as bone morphogenetic proteins (BMPs) and glial cell derived neurotrophic factor (GDNF). Many of these proteins are known to play pivotal roles in tumor progression, invasion, and metastasis. TGF-*β* has been previously shown to increase cell motility and *α*v*β*3 integrin expression in human chondrosarcoma cells, via pathways involving PI3K, Akt, and NF-*κ*B [53]. BMPs are proteins originally isolated from bone tissue, and are capable of ectopically inducing new cartilage and bone formation. BMP-2 has been found to act through PI3K/Akt, IKK*α*/*β*, and NF-*κ*B, resulting in increased *β*1 integrin expression and migration of human chondrosarcoma cells [54]. GDNF is a factor required for survival, proliferation, and activation of glioma cells. GDNF has been shown to promote the migration of human chondrosarcoma cells by upregulating *α*v*β*3 integrin expression, through activation of the MEK/ERK, IKK*α*/*β*, and NF-*κ*B pathways [55]. A novel cytokine system, consisting of receptor activator of NF-*κ*B ligand (RANKL), its receptor, RANK, and the protein osteoprotegerin (OPG), has been identified and extensively characterized for its role in bone remodeling. The RANKL/RANK signaling axis has also been found to regulate cell migration in human chondrosarcoma cells, through MEK, ERK, IKK*α*/*β*, and NF-*κ*B signaling and upregulation of *β*1 integrin [56] (Table 1).

Certain inflammation-related molecules may also play important roles in regulating migration in human chondrosarcoma cells. Cyclooxygenase-2 (COX-2), an inducible enzyme that catalyzes the formation of prostaglandin E2 (PGE_2_) during inflammation, is one such molecule. PGE_2_ appears to upregulate the expression of the *α*2*β*1 integrin via the EP1/PLC/PKCa/c-Src signaling pathways, leading to increased cell migration [57]. Bradykinin (BK) is a vasoactive peptide that mediates inflammatory responses and can also stimulate cell proliferation. BK was found to enhance chondrosarcoma cell migration by increasing *α*2*β*1 integrin expression, through the BK receptor and PLC/PKC*δ*/NF-*κ*B signal transduction pathways [58]. High mobility group box chromosomal protein 1 (HMGB-1) was originally identified as a nuclear protein that plays important roles in chromatin organization and transcriptional regulation. HMGB-1 has multiple functions, including the release of pro-inflammatory cytokines, cell proliferation, and cell migration. In human chondrosarcoma cells, HMGB-1 appears to promote cell migration by increasing *α*v*β*1 integrin expression, through the RAGE (receptor for advanced glycation end products)/PI3 K/Akt/c-Jun/AP-1 signal transduction pathway [59] (Table 1).

## 5. Integrins as Signaling Receptors Regulating Chondrosarcoma Progression

High levels of integrin expression have been found in chondrosarcomas. Often, this is correlated with metastasis and poor prognosis. In light of this, it is noteworthy that integrins can regulate a wide range of signaling pathways critical for tumor growth and metastasis.

Increasing evidence suggests that ECM and its degradation products could play important roles in cancer progression and metastasis. Many of the underlying mechanisms are likely to involve integrin signaling. Proteomic comparison of human chondrogenic tumors revealed that the protein C-propeptides of procollagens I*α*1 (PC1CP) were highly expressed in human chondrosarcomas, but not in benign enchondromas. Soluble PC1CP can induce the expression of VEGF and CXCR4 in a *β*1 integrin-dependent manner, and this has been linked to chondrogenic tumor vascularization and progression [60]. On the other hand, a different extracellular matrix protein, the NH_2_-propeptide of type IIB procollagen (PIIBNP), was found to be capable of inducing cell death in chondrosarcoma, cervical and breast cancer cell lines, via its interaction with the integrins *α*v*β*3 and *α*v*β*5 [61]. Osteopontin (OPN) is an important component of the extracellular matrix in bone. The OPN protein has also been found to play a crucial role in determining the metastatic potential of various cancers. For example, OPN enhances the migration of chondrosarcoma cells by upregulating MMP-9 expression, through the *α*v*β*3 integrin receptor, FAK (Focal Adhesion Kinase), MEK, ERK, and NF-*κ*B-dependent signaling pathways [62, 63] (Table 2).

The CCN family of small secreted cysteine-rich proteins has six members (CCN1 to CCN6). The name CCN is derived from the first three members of the family to be discovered, namely, CYR61 (cysteine-rich angiogenic protein 61 or CCN1), connective tissue growth factor (CTGF/CCN2), and nephroblastoma overexpressed (NOV/CCN3). CCNs appear to regulate numerous biological processes, such as differentiation, migration, proliferation, and cell adhesion. Notably, aberrant expression of CCNs has been identified in a broad range of tumor types. In human chondrosarcoma cells, CCN1, CCN2, and CCN3 have been found to enhance cell migration by increasing MMP-13 expression; this is mediated via the *α*v*β*3 integrin receptor and FAK-dependent signaling mechanisms [64–66]. Other members of the CCN family have also been studied, including CCN4 (WISP-1) and CCN6 (WISP-3). These integrin-binding proteins appear to regulate cell migration in human chondrosarcoma cells by inducing integrin-dependent signaling. CCN4 (WISP-1) increases the activity of MMP-2, via the *α*v*β*1 integrin receptor and the FAK, MEK, ERK, IKK*α*/*β*, and NF-*κ*B pathways, leading to enhanced migration of human chondrosarcoma cells [73]. Likewise, CCN6 (WISP-3) appears to function by increasing ICAM-1 expression through the *α*v*β*3 and *α*v*β*5 integrin receptor, FAK, MEK, ERK, c-Jun, and AP-1 pathways [74] (Table 2).

## 6. Integrins as Therapeutic Targets in Chondrosarcomas

Given the important roles of integrin-mediated signaling in metastasis and cancer progression, there has been increasing interest in therapeutic strategies to target these proteins. In human chondrosarcomas, increased expression of integrins, including *α*2*β*1, *α*v*β*1, *α*v*β*3, *β*1, and *β*5, is closely associated with tumor progression and metastasis. Signaling through integrin receptors, such as *α*v*β*1, *α*v*β*3, *α*v*β*5, and *β*1, may also promote cancer progression by regulating cell migration. This review discusses a selection of emerging therapeutic approaches for chondrosarcoma, together with their underlying molecular mechanisms. These include (i) integrin antagonists, (ii) inhibition of the RANK/RANKL/OPG axis, (iii) inhibition of FAK, (iv) inhibition of the IGF-I/IGF-IR axis, and (v) herbal medicines.

### 6.1. Integrin Antagonists

Since the discovery of the integrin-binding RGD sequence motif and its importance in mediating cell attachment, efforts have been made to develop RGD-related small molecules as integrin antagonists. Cilengitide, a cyclic RGD pentapeptide, is the first antiangiogenic small molecule developed to target the integrins *α*v*β*3, *α*v*β*5, and *α*v*β*1 [75]. This drug is currently being tested in phase III clinical trials for treatment of glioblastomas, and in phase II trials for several other tumor types [76]. Chemical modifications to the cilengitide molecule, including N-methylation at distinct positions, can modulate its biological, structural, and pharmacokinetic properties; this could enhance selectivity, particularly for the *α*v*β*3 subtype [77]. In addition, since integrin *α*v*β*3 is expressed on the blood vessels that supply tumors, as well as on the tumor cells themselves, antagonists to this integrin might be particularly useful for treatment of chondrosarcoma. Another drug, ATN-161, is a non-RGD-based peptide inhibitor of *α*v*β*1 that is currently in clinical trials for cancer. In patients with advanced solid tumors who were given ATN-161, prolonged stable disease was observed in up to a third of the patients [78]. In a murine model of metastatic colorectal cancer, combination therapy with ATN-161 and 5-fluorouracil was found to reduce metastasis and improve survival [79]. ATN-161 has also been shown to reduce growth and metastasis of breast cancer cells implanted in mice [80]. The above findings suggest that this *α*v*β*1-inhibiting drug holds promise for the treatment of human chondrosarcomas.

Etaracizumab (also known as vitaxin, Abegrin, or MEDI-522), a humanized anti-*α*v*β*3 antibody, was the first anti-integrin monoclonal antibody to be tested in clinical trials for cancer. In a phase I study on etaracizumab, prolonged disease stabilization was observed in a number of cancer patients with metastatic lesions, who received the drug beyond the first cycle of therapy [81]. Etaracizumab was also shown to decrease osteoclastic bone resorption by impairing osteoclast attachment, without affecting osteoclast formation and multinucleation; this could be useful for reducing metastatic bone loss in cancer patients [82]. Volociximab (M200) is a chimeric mouse-human anti-*α*v*β*1 monoclonal antibody, which has shown anti-angiogenic activity *in vitro* and *in vivo* [83, 84]. In clinical trials, volociximab was well tolerated, and there is support for its efficacy in metastatic melanoma and renal cell carcinoma [85]. Consequently, these integrin antagonists may also have therapeutic potential for chondrosarcomas, to reduce metastasis and control tumor progression.

The small molecule compound L-000845704 is an orally bioavailable nonpeptide *α*v*β*3 antagonist, which has been tested in preclinical and clinical trials for the treatment of osteoporosis [86, 87]. Another orally active nonpeptide *α*v*β*3 antagonist, SB 273005, has been shown to prevent and reduce edema and inflammation in a rat model of adjuvant-induced arthritis [88]. The potential applications of these integrin antagonists in the treatment of chondrosarcomas could be explored.

### 6.2. Inhibition of the RANK/RANKL/OPG Axis

In human chondrosarcoma tissues, RANKL and RANK expressions are higher than those in normal cartilage. Activation of the RANK/RANKL axis leads to the upregulation of *β*1 integrin, and contributes to enhanced migration in human chondrosarcoma cells [56]. These observations have prompted efforts to develop therapies targeting RANKL. One promising approach involves the targeting of RANKL signaling with a decoy receptor, OPG, or with a soluble receptor form (RANK-Fc); this has been shown to inhibit bone metastasis in a number of murine models [89–91]. A number of clinical trials involving denosumab, a fully human monoclonal antibody against RANKL, support its use as an alternative treatment option for bone metastases [92–94].

### 6.3. Inhibition of FAK (Focal Adhesion Kinase)

Numerous studies indicate that integrin signaling through FAK plays a role in promoting migration of chondrosarcoma cells [62, 64–66, 73, 74, 95]. Inhibition of endogenous FAK activity by adenoviral overexpression of the C-terminal domain of FAK effectively interrupts FAK signaling and its downstream events; this was found to decrease cell invasiveness in chondrosarcoma cell lines [96]. A recent phase I trial of an inhibitor of FAK showed antitumor efficacy and minimal toxicity in patients with advanced solid tumors. Such results indicate that FAK might be another promising therapeutic target [97].

### 6.4. Inhibition of the IGF-I/IGF-IR Axis

Insulin-like growth factor 1 (IGF-1) can enhance the migration of chondrosarcoma cells by upregulating integrin expression. In addition, integrin binding can also regulate IGF-1 receptor (IGF-1R) signaling [98]. Consistent with this, blocking ligand occupancy of integrins reduced IGF-1-stimulated receptor phosphorylation, and inhibited cellular migration and DNA synthesis in response to IGF-1 [99]. This suggests that the IGF-1 signaling pathway may be another potential therapeutic target in chondrosarcoma [100]. Various IGF-1R monoclonal antibodies, including R1507, figitumumab, and ganitumab (AMG 479), have emerged as promising drugs for the treatment of Ewing's sarcoma, a small round-cell tumor that typically arises in the bones and soft tissues. A number of clinical trials to test these novel therapies are ongoing [101–105]. These drugs could potentially be explored for the treatment of other sarcomas, including chondrosarcoma.

### 6.5. Herbal Medicine

Berberine, an isoquinoline alkaloid, is a bioactive molecule found in the Ranunculaceae and Papaveraceae plant families. Berberine, which has been shown to inhibit cancer cell migration, was shown to downregulate *α*v*β*3 integrin expression through the PKC*δ*, c-Src, and AP-1 pathways [106].

## 7. Conclusion

**Figure 1:**
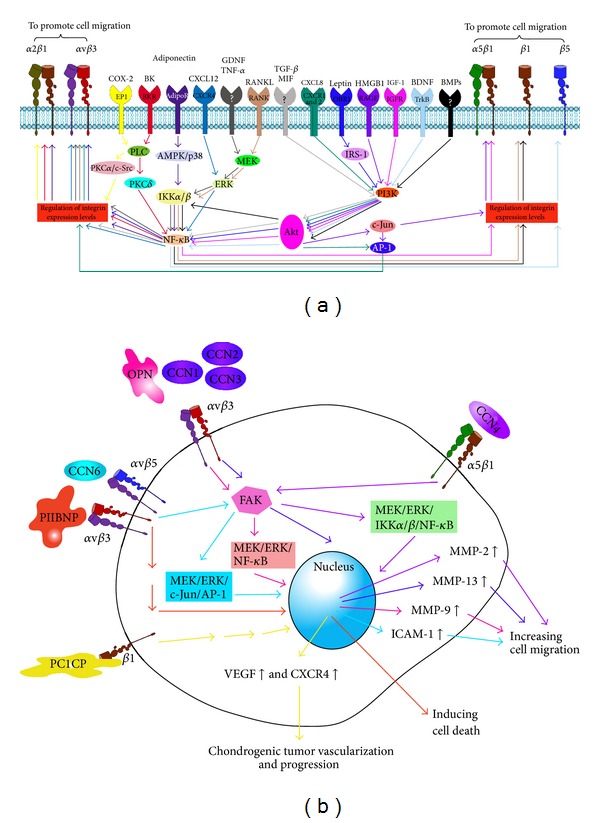
Schematic representation of the mediators that increase surface expression of integrin (a) and integrin-mediated signalings (b) which are shown to be novel therapeutic targets for chondrosarcomas.

Chondrosarcomas are the second most common form of bone malignancy. These tumors are relatively resistant to chemotherapy and radiotherapy; currently, surgical resection is the only effective therapeutic option. However, 5–10% of conventional chondrosarcomas are high-grade tumors, which show high metastatic potential and poor outcomes after resection alone. It is therefore crucial to identify and develop effective adjuvant treatments. Integrins, which are cell surface proteins involved in diverse biological processes, have been implicated in cancer cell migration, invasion, and metastasis, during tumor progression. Consequently, targeting of integrin expression and signaling has been considered a promising approach in cancer therapy. Nevertheless, integrins play a crucial role in many physiological processes; for example, tissue morphogenesis, inflammation, wound healing, and regulation of cell growth and differentiation. Any inhibition of these may cause serious adverse effects that must be taken to into account. Clinical and preclinical studies aimed at inhibiting integrin expression and signaling are ongoing. To date, however, integrin-targeted therapeutics in chondrosarcomas have not yet been successfully translated into clinical practice. This review summarizes recent progress in elucidating the molecular basis for integrin function in cancer. We have discussed various mechanisms and mediators that regulate the expression of integrins and integrin-mediated signaling (Figure 1). This understanding of molecular mechanisms could be translated into effective therapies for chondrosarcoma.

## Figures and Tables

**Table 1:** Regulation of integrin expression in human chondrosarcoma cells.

Groups	Activators	Integrins	Pathway	References
Growth factors	Insulin-like growth factor-I (IGF-I)	*α*5*β*1	IGF-I receptor/PI3K/Akt/NF-*κ*B	[44]
	Brain derived neurotrophic factor (BDNF)	*β*5	TrkB receptor/PI3K/Akt/NF-*κ*B	[45]

Chemokines	IL-8/CXCL8	*α*v*β*3	CXCR1 and CXCR2/PI3K/Akt/AP-1	[46]
	CXCL12/SDF-1	*α*v*β*3	CXCR4/ERK/NF-*κ*B	[47]

Pro-inflammatory cytokines	TNF-*α*	*α*v*β*3	MEK/ERK/IKK*α*/*β*/NF-*κ*B	[48]
	Leptin	*α*v*β*3	OBR1/IRS-1/PI3K/Akt/NF-*κ*B	[49]
	Adiponectin	*α*2*β*1	AdipoR/AMPK/p38/IKK*α*/*β*/NF-*κ*B	[50]
	Macrophage migration-inhibitory factor (MIF)	*α*v*β*3	PI3K/Akt/NF-*κ*B	[51]

Cytokines	TGF-*β*	*α*v*β*3	PI3K/Akt/NF-*κ*B	[52]
	Bone morphogenetic proteins (BMPs)	*β*1	PI3K/Akt/IKK*α*/*β*/NF-*κ*B	[53]
	Glial cell derived neurotrophic factor (GDNF)	*α*v*β*3	MEK/ERK/IKK*α*/*β*/NF-*κ*B	[54]
	Receptor activator of nuclear factor kappa-B ligand (RANKL)	*β*1	RANK/MEK/ERK/IKK*α*/*β*/NF-*κ*B	[55]

Inflammatory-related molecules	Cyclooxygenase-2 (COX-2)	*α*2*β*1	EP1/PLC/PKC*α*/c-Src	[56]
	Bradykinin (BK)	*α*2*β*1	BK receptors/PLC/PKC*δ*/NF-*κ*B	[57]
	High mobility group box chromosomal protein 1 (HMGB1)	*α*5*β*1	RAGE (receptor for advanced glycation end products)/PI3K/Akt/c-Jun/AP-1	[58]

**Table 2:** Integrin as a receptor regulates signalings in human chondrosarcoma cells.

Groups	Ligand	Integrin signaling	Regulation	Function	References
Extracellular matrix and its degradation fragments and by-products	PC1CP	*β*1	VEGF expression ↑ CXCR4 expression ↑	Inducing chondrogenic tumor vascularization and progression	[59]
	PIIBNP	*α*v*β*3 and *α*v*β*5		Inducing cell death	[60]
	OPN	*α*v*β*3/FAK/MEK/ERK/NF-*κ*B	MMP-9 expression ↑	Increasing cell migration	[61]

CCN family	CCN1	*α*v*β*3/FAK	MMP-13 expression ↑	Increasing cell migration	[62]
	CCN2	*α*v*β*3/FAK	MMP-13 expression ↑	Increasing cell migration	[63]
	CCN3	*α*v*β*3/FAK	MMP-13 expression ↑	Increasing cell migration	[64]
	CCN4	*α*5*β*1/FAK/MEK/ERK/IKK*α*/*β*/NF-*κ*B	MMP-2 activity ↑	Increasing cell migration	[65]
	CCN6	*α*v*β*3 and *α*v*β*5/FAK/MEK/ERK/c-Jun/AP-1	ICAM-1 expression ↑	Increasing cell migration	[66]
